# Limitations of Artificial Intelligence Generated Images for Hand Surgery Patient Education

**DOI:** 10.1016/j.jhsg.2025.100845

**Published:** 2025-10-10

**Authors:** Jessica L. Duggan, Omar Mohamed, Euan Forrest, Jessica Guo, Tamara D. Rozental

**Affiliations:** ∗ Harvard Combined Orthopaedic Residency Program, Boston, MA; † Department of Hand and Upper Extremity Surgery, Beth Israel Deaconess Medical Center, Boston, MA; ‡ Harvard Medical School, Boston, MA

**Keywords:** AI, Artificial intelligence, Hand surgery, Patient education, Text-to-image generation

## Abstract

**Purpose:**

The role of artificial intelligence (AI) in medicine is rapidly evolving, with potential to improve both the clinician and patient experience. We sought to evaluate whether popular AI text-to-image generators could create anatomically accurate images of common hand surgery procedures. We hypothesized that the AI-generated images would not be adequate as patient education materials.

**Methods:**

We queried five AI text-to-image generators: Craiyon, DALL-E, DeepSeek, Gemini, Midjourney, and Stable Diffusion. They were given the prompt, “Create an anatomically accurate image with labels of [Condition] surgical approach to be used as a visual aid for patient education,” with the following conditions inserted: carpal tunnel syndrome, Dupuytren contracture, trigger finger, thumb carpometacarpal arthritis, and de Quervain tenosynovitis. Images were then graded on legibility, detail and clarity, anatomical realism and accuracy, appropriate surgical site, and lack of fabricated anatomy. Images could score a maximum of 2 points per each criterion, with an assumed Control score of 10 points.

**Results:**

A total of 1,500 images were generated and reviewed. When comparing total scores, all AI generators performed significantly lower than the Control, except for DALL-E’s images of Dupuytren contracture. For the image detail and clarity category, DALL-E, DeepSeek, Gemini, and Midjourney all scored similarly to the Control and each other. For the remaining criteria (legibility, anatomic realism, surgical site, fabricated anatomy), each of the AI generators scored significantly lower than the Control generator. In total, 99.8% of images contained at least some degree of fabricated anatomy. DALL-E consistently had the highest scores for each category, while Craiyon had the lowest.

**Conclusions:**

Although the AI servers successfully produced highly detailed and visually engaging images, they failed to portray accurate anatomy and often included fictitious structures. Further work is needed to train and fine tune AI models to produce accurate and appropriate images.

**Type of study/level of evidence:**

Therapeutic V.

Over the past several years, artificial intelligence (AI) text-to-image generators have rapidly grown in popularity and their application to various fields. In medicine, AI-generated images may potentially enhance diagnostics and therapeutics, create large image databases, and assist in trainee and patient education.^1^, ^2^, ^3^ For example, several popular image generators (DALL-E, Stable Diffusion, and Craiyon) have been studied as a new tool for enhancing anatomic images and diagrams used in medical education.^4^ Image generators have also been used to evaluate the diversity and demographics of physicians depicted by AI, including in internal medicine, cardiology, plastic surgery, orthopedic surgery, general surgery, and hand surgery.^5^, ^6^, ^7^, ^8^, ^9^, ^10^

In addition to targeting clinicians and trainees, AI-generated images may serve to improve the patient experience. The utility of visual aids for patients undergoing surgery has been well documented: visuals improve patient knowledge recall, increase patient satisfaction, decrease their anxiety, and facilitate the shared decision making and consent process.^11^, ^12^, ^13^ Similar findings have been published in hand surgery, with patients undergoing knee arthroscopy demonstrating better information recall, 6-week retention, and overall satisfaction when given a multimedia presentation during the consent process when compared to standard verbal consent.^14^

Notably, it is not solely the presence of visual materials, but quality and appropriateness for their target audiences, that provides benefit to patients. Evaluations have shown that photos and drawings from textbooks and academic journals are too complex; these images hinder overall readability of patient education materials.^15^ Prior work investigating patient perspectives indicate that images presented in public health infographics often lack clarity and fail to emphasize their messages with associated symbols or text.^16^ Thus, there may be a role for AI to generate images that are informative, yet accessible to patient audiences.

Although work has been performed to evaluate AI’s ability to create or bolster written patient education materials for hand surgery, few if any studies have explored use of AI image generators for this purpose.^3^^,^^17^ Here, we sought to evaluate and compare the abilities of popular AI text-to-image generators to create anatomically accurate depictions of hand surgery that could be incorporated into patient education materials. We hypothesized that AI image generators currently cannot reliably create accurate images that would be adequate for patient consumption.

## Materials and Methods

### Study design

We performed a qualitative and comparative study of test images produced by the following popular AI text-to-image generators: Craiyon, DALL-E, DeepSeek, Gemini, Midjourney, and Stable Diffusion. These six servers were chosen based on user accessibility, image photorealism, and image accuracy.^18^, ^19^, ^20^ We ensured to include servers that portrayed greater diversity in their images, as noted in prior work investigating applications of AI to the surgical subspecialties.^8^^,^^9^ Institutional Review Board approval was not required for this study.

### Image collection

We sought to collect AI-generated images of the surgical approaches for the following five pathologies commonly seen in the hand surgery clinic: carpal tunnel syndrome (CTS), Dupuytren contracture (DC), trigger finger (TF), thumb carpometacarpal arthritis (CMC), and de Quervain tenosynovitis (DQ). To obtain each image, the AI models were all given the prompt, “Create an anatomically accurate image with labels of [Condition] surgical approach to be used as a visual aid for patient education,” with each of the various hand conditions being pasted into the prompt. The searches were repeated 50 times per condition per AI server (see below for power analysis). Each search was performed in a new conversation window to avoid iterative responses and allow each image generation to be unbiased by the previous ones.

### Assessment of AI-generated images

**Figure 1:**
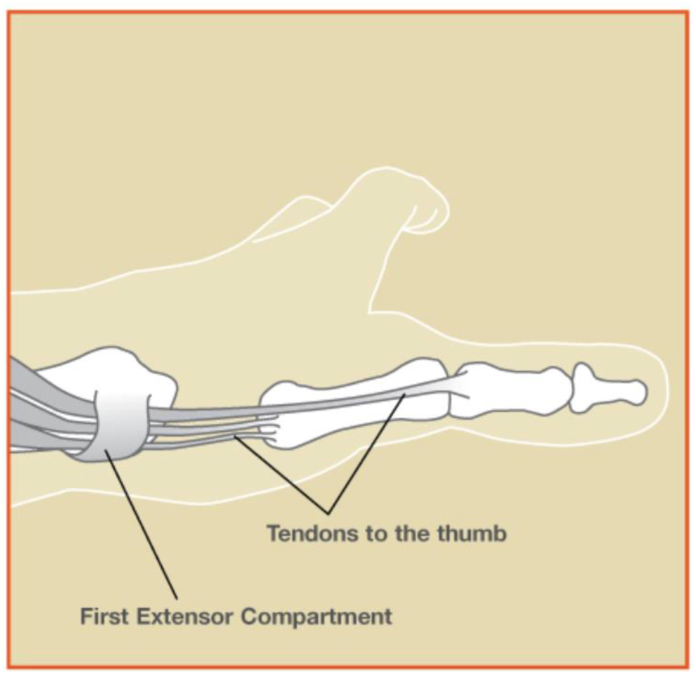
Sample Control image from the Handcare online resource created by the American Society for Surgery of the Hand.^23^ Completely meets all criteria evaluated in this study (legibility, detail and clarity, anatomical realism and accuracy, appropriate surgical site, and lack of fabricated anatomy). Reproduced with permission from American Society for Surgery of the Hand.

Following image collection, each image was reviewed by three independent reviewers (hand surgery fellow, hand surgery resident, and medical student). An attending surgeon reviewed the grading criteria and image evaluation results. Images were evaluated on the following criteria – legibility, image detail and clarity, anatomical realism and accuracy, appropriate surgical site, and lack of fabricated anatomy. These criteria were modeled after the grading system used by Moin et al^21^ in evaluating AI-generated medical illustrations of corneal transplant surgery and prior studies that assessed health care related visual materials for both patient and surgical trainee education.^16^^,^^22^ For each criterion, the image could score zero, one, or two points, with a score of zero indicating failure to meet criteria, one indicating partially meeting criteria, and two indicating completely meeting criteria (Table 1). Scores were averaged across the three reviewers to give a “final” score to each AI generator in each criterion assessed. We then compared the scores of each image generator to each other and to an assumed “Control” generator score of 10 (ie, completely meeting all criteria). A sample Control image (ie, that would score 10/10 based on our criteria) from the Handcare resources created by the American Society for Surgery of the Hand is shown in Figure 1 for reference.^23^

**Table 1:** Grading System Used to Evaluate each AI-generated Image

Legibility (0–2) 0: all labels illegible or not present (spelling/visual errors throughout) 1: some labels illegible (some errors) 2: all labels are legible (no errors)
Image detail and clarity (0–2) (Not a grade of realism or accuracy) 0: image is blurry, unclear, and lacks detail 1: image has some details, but some areas are unclear 2: image is detailed and clear
Anatomical realism and accuracy (0–2) 0: no critical structures are shown 1: critical structures shown, but incorrectly positioned 2: all critical structures shown and correctly positioned
Appropriate surgical site (0–2) 0: site not depicted (eg, dorsal hand for carpal tunnel release) 1: site present in image, but not emphasized/centered 2: appropriate surgical site depicted and emphasized (colors, positioning)
Lack of fabricated anatomy (0–2) 0: significant fictitious structures throughout image, distracts from overall image 1: some fictitious structures that do not distract from overall image 2: anatomic structures are legitimate

### Statistical analysis

Descriptive statistics were obtained using Microsoft Excel (Microsoft, Redmond, WA). Statistical analyses were performed using R Studio (version 2024.12.0+467). Given our grading system yielded ordinal dependent variables that were assumed to be non-normally distributed, we chose to use the Kruskal–Wallis H test for statistical comparison. An a priori power analysis calculated that 39.2 images would be required to detect a medium effect size of 0.25 using this test, and we collected sufficient images to meet this criterion. Interobserver reliability was calculated using Fleiss’ kappa analysis. A significance level of 0.05 was used throughout.

## Results

**Figure 2:**
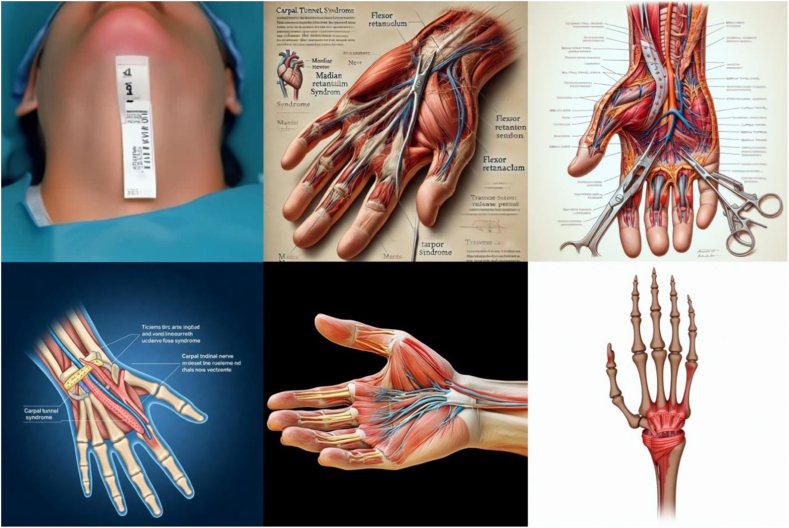
Representative image outputs in response to the query, “Create an anatomically accurate image with labels of CTS surgical approach to be used as a visual aid for patient education.” Top left: Craiyon, top middle: DALL-E, top right: DeepSeek, bottom left: Gemini, bottom middle: Midjourney, bottom right: Stable Diffusion.

**Figure 3:**
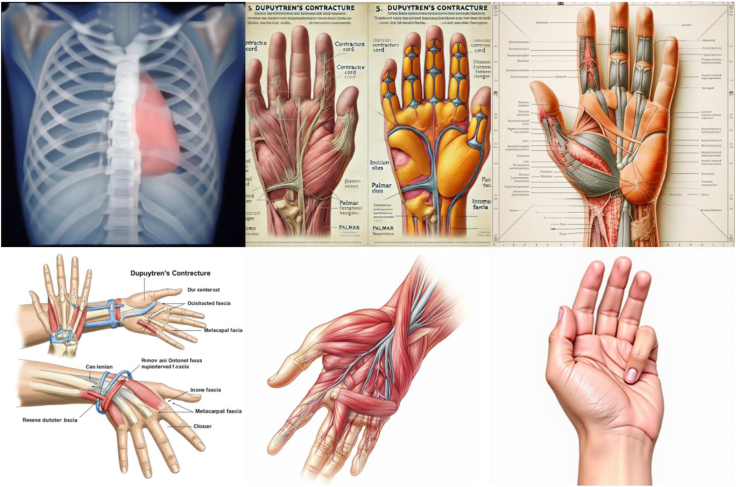
Representative image outputs in response to the query, “Create an anatomically accurate image with labels of Dupuytren contracture surgical approach to be used as a visual aid for patient education.” Top left: Craiyon, top middle: DALL-E, top right: DeepSeek, bottom left: Gemini, bottom middle: Midjourney, bottom right: Stable Diffusion.

**Figure 4:**
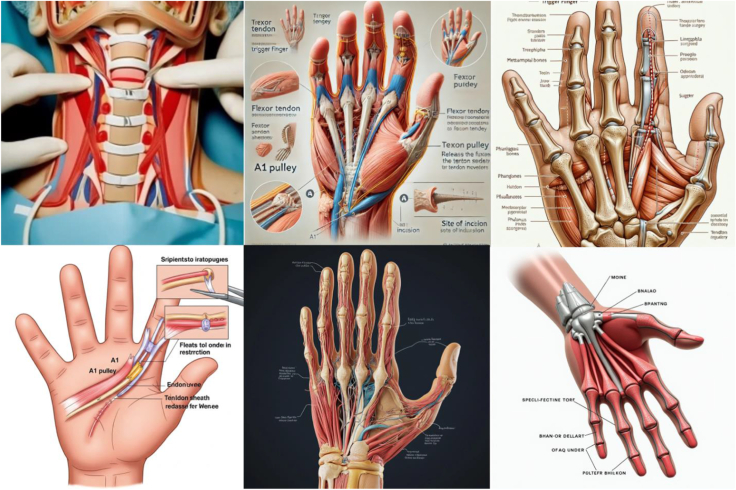
Representative image outputs in response to the query, “Create an anatomically accurate image with labels of TF surgical approach to be used as a visual aid for patient education.” Top left: Craiyon, top middle: DALL-E, top right: DeepSeek, bottom left: Gemini, bottom middle: Midjourney, bottom right: Stable Diffusion.

**Figure 5:**
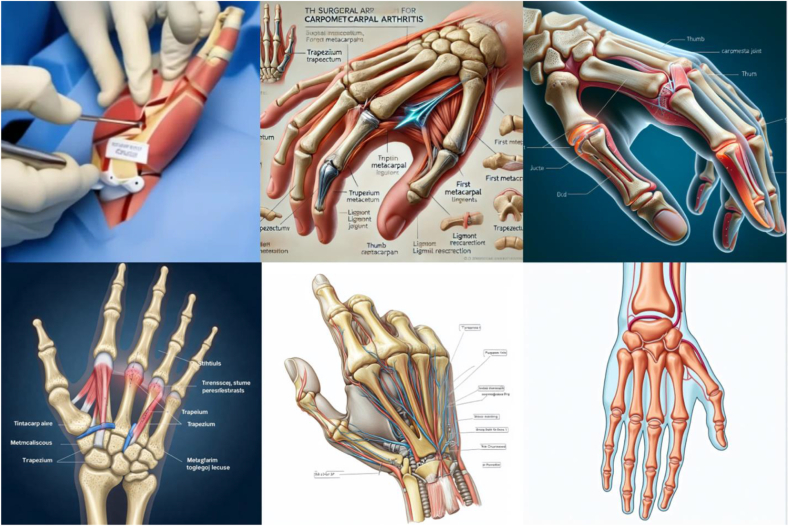
Representative image outputs in response to the query, “Create an anatomically accurate image with labels of thumb carpometacarpal arthritis surgical approach to be used as a visual aid for patient education.” Top left: Craiyon, top middle: DALL-E, top right: DeepSeek, bottom left: Gemini, bottom middle: Midjourney, bottom right: Stable Diffusion.

**Figure 6:**
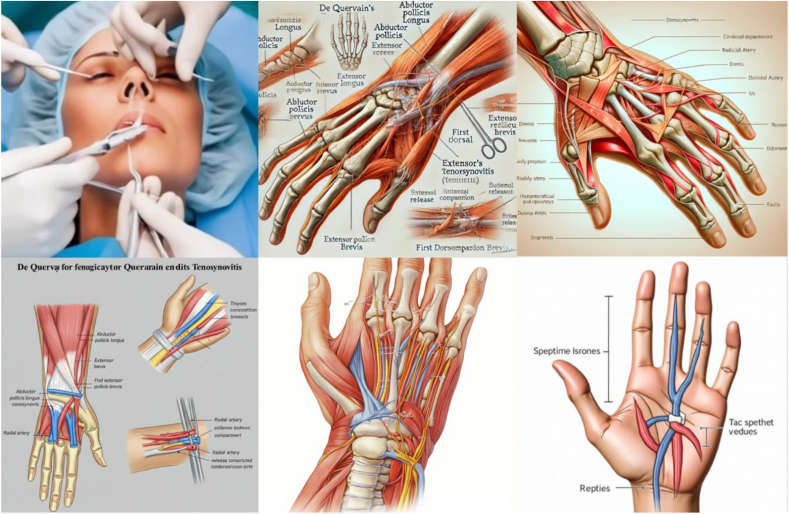
Representative image outputs in response to the query, “Create an anatomically accurate image with labels of de Quervain tenosynovitis surgical approach to be used as a visual aid for patient education.” Top left: Craiyon, top middle: DALL-E, top right: DeepSeek, bottom left: Gemini, bottom middle: Midjourney, bottom right: Stable Diffusion.

A total of 1,500 images (5 procedures × 6 generators × 50 queries each) were generated using the standardized prompts and graded by the three independent reviewers. Fleiss’ kappa was 0.772, demonstrating “substantial” agreement across reviewers. Samples of the images generated for each procedure are displayed in Figure 2, Figure 3, Figure 4, Figure 5, Figure 6. There was no considerable variation in the 50 images produced by each server for any condition.

### Overall performance

For CTS, the mean total scores for each AI generator were as follows: 1.48 ± 0.85 for Craiyon, 4.99 ± 1.31 for DALL-E, 3.80 ± 1.20 for DeepSeek:, 4.07 ± 1.40 for Gemini, 2.9 3 ± 0.91 for Midjourney, and 2.76 ± 0.75 for Stable Diffusion. AI generators all scored lower than the Control image generator (*P* < .001). DALL-E and Gemini each scored significantly higher than Craiyon, Midjourney, and Stable Diffusion (*P* < .02). Craiyon scored significantly lower than all other generators (*P* < .002).

For Dupuytren contracture, the mean total scores for each AI generator were as follows: 1.75 ± 0.75 for Craiyon, 6.87 ± 1.35 for DALL-E, 3.68 ± 1.45 for DeepSeek:, 3.36 ± 0.83 for Gemini, 2.57 ± 0.60 for Midjourney, and 2.88 ± 1.20 for Stable Diffusion. AI generators all scored lower than the Control generator (*P* < .001), except for DALL-E (*P* = .10). DALL-E scored significantly higher than all other generators (*P* < .001).

For TF, the mean total scores for each AI generator were as follows: 1.72 ± 0.68 for Craiyon, 5.38 ± 1.33 for DALL-E, 4.03 ± 1.31 for DeepSeek, 3.69 ± 1.18 for Gemini, 2.51 ± 0.35 for Midjourney, and 2.62 ± 0.46 for Stable Diffusion. AI generators all scored lower than the Control generator (*P* < .01). DALL-E, DeepSeek, and Gemini each scored significantly higher than Craiyon, Midjourney, and Stable Diffusion (*P* < .007).

For thumb carpometacarpal arthritis, the mean total scores for each AI generator were as follows: 2.01 ± 1.23 for Craiyon, 6.01 ± 0.57 for DALL-E, 5.15 ± 1.01 for DeepSeek, 5.81 ± 0.75 for Gemini, 4.15 ± 0.73 for Midjourney, and 5.00 ± 0.67 for Stable Diffusion. AI generators all scored lower than the Control generator (*P* < .001). DALL-E and Gemini each scored significantly higher than Craiyon, Midjourney, and Stable Diffusion (*P* < .03). Craiyon scored significantly lower than all other generators (*P* < .05).

For de Quervain tenosynovitis, the mean total scores for each AI generator were as follows: 1.41 ± 0.50 for Craiyon, 5.47 ± 0.68 for DALL-E, 4.05 ± 0.91 for DeepSeek, 4.53 ± 1.09 for Gemini, 3.86 ± 0.81 for Midjourney, and 2.63 ± 0.68 for Stable Diffusion. AI generators all scored lower than the Control generator (*P* < .01). DALL-E outperformed all other AI generators (*P* < .001), except for Gemini (*P* = .12) (Table 2).

**Table 2:** Mean Total Scores for Control, Craiyon, DALL-E, DeepSeek, Gemini, Midjourney, and Stable Diffusion, With a Maximum Score of 10 Points.

Pathology	Control	Craiyon	DALL-E	DeepSeek	Gemini	Midjourney	Stable Diffusion	P Value∗
CTS	10.00	1.48 ± 0.85	4.99 ± 1.31	3.80 ± 1.20	4.07 ± 1.40	2.93 ± 0.91	2.76 ± 0.75	<.001
DC	10.00	1.75 ± 0.75	6.87 ± 1.35	3.68 ± 1.45	3.36 ± 0.83	2.57 ± 0.60	2.88 ± 1.20	<.001
TF	10.00	1.72 ± 0.68	5.38 ± 1.33	4.03 ± 1.31	3.69 ± 1.18	2.51 ± 0.35	2.62 ± 0.46	<.001
CMC arthritis	10.00	2.01 ± 1.23	6.01 ± 0.57	5.15 ± 1.01	5.81 ± 0.75	4.15 ± 0.73	5.00 ± 0.67	<.001
DQ	10.00	1.41 ± 0.50	5.47 ± 0.68	4.05 ± 0.91	4.53 ± 1.09	3.86 ± 0.81	2.63 ± 0.68	<.001

Values presented as mean ± standard deviation.

∗ *P* values comparing entire cohort of AI generators to Control generator.

### Performance on individual grading categories

In every grading category (legibility, anatomic realism and accuracy, appropriate surgical site, lack of fabricated anatomy) except image detail and clarity, each of the AI generators scored significantly lower than the Control generator (*P* < .001). For the legibility category, DALL-E (0.89 ± 0.28) performed the best, scoring significantly higher than all other generators (*P* < .001), followed by Gemini (0.39 ± 0.43), then DeepSeek (0.18 ± 0.33). For the image detail and clarity category, DALL-E, DeepSeek, Gemini, and Midjourney (2.00 ± 0.00, 1.99 ± 0.09, 1.99 ± 0.07, and 1.99 ± 0.05, respectively) all scored similarly to the Control generator and each other (*P* = 1.00). Craiyon and Stable Diffusion (1.14 ± 0.35 and 1.82 ± 0.31, respectively) both scored significantly lower than both the Control generator and the other AI generators (*P* < .001). For the anatomical realism and accuracy category, DALL-E (0.80 ± 0.65) scored significantly higher than all other generators (*P* < .001). Gemini and DeepSeek (0.54 ± 0.57 and 0.53 ± 0.59, respectively) both scored significantly higher than Craiyon, Midjourney, and Stable Diffusion (*P* < .003). Craiyon (0.06 ± 0.23) scored lower than all other generators (*P* < .003).

**Figure 7:**
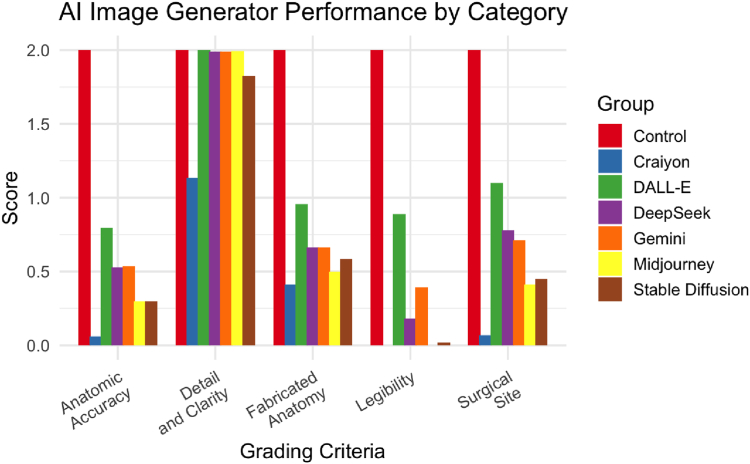
Comparison of mean scores in each grading criteria for Control and all AI image generators.

For the appropriate surgical site category, DALL-E (1.10 ± 0.71) scored significantly higher than all other generators (*P* < .001). Gemini and DeepSeek (0.71 ± 0.58 and 0.78 ± 0.68, respectively) both scored significantly higher than Craiyon, Midjourney, and Stable Diffusion (*P* < .001). Craiyon (0.07 ± 0.25) scored lower than all other generators (*P* < .001). For the lack of fabricated anatomy category, 1,497 (99.8%) images contained at least some fictitious structures. DALL-E (0.96 ± 0.16) scored significantly higher than all other generators (*P* < .001). Craiyon and Midjourney (0.41 ± 0.46 and 0.50 ± 0.37, respectively) each scored significantly lower than all other generators (*P* = .001) (Fig 7).

### Comparison across procedures

When comparing the combined AI generator performance across the different procedures queried, the mean total scores for thumb CMC arthritis were significantly higher than all other procedures (*P* < .001). Broken down by grading criteria, the thumb CMC arthritis images scored significantly higher on anatomical realism and accuracy and appropriate surgical site when compared to the remaining four procedures. There were no differences in lack of fabricated anatomy across the different procedures.

## Discussion

The applications of AI in medicine are rapidly evolving and offer great potential for producing outputs in an efficient, accessible, and interactive manner for both clinicians and patients. In hand surgery, prior studies have investigated AI algorithms to detect fractures, diagnose common hand conditions through medical history, and answer frequently asked questions.^24^, ^25^, ^26^ Although emphasis has been placed on AI chat bots and large language models, little work has been performed evaluating the use of AI text-to-image generators to enhance the patient experience. Our study evaluated the performance of five popular AI image generators in producing patient-friendly visual aids for common hand procedures.

Our main findings include the following: (1) When assessing the overall scores for each hand condition, all AI generators performed significantly lower than the Control, with the exception of DALL-E’s images of Dupuytren contracture. (2) When broken down by grading criteria, each of the AI generators scored significantly lower than the Control generator in all categories (legibility, anatomic realism and accuracy, appropriate surgical site, lack of fabricated anatomy), except for image detail and clarity. (3) DALL-E consistently had the highest scores for each category, whereas Craiyon had the lowest. (4) When comparing across procedure queried, scores for the thumb CMC arthritis were significantly higher than all other procedures.

Our data supports the ability of AI image generators to produce highly detailed, visually engaging images in a matter of seconds, as four out of sic generators used scored on par with the Control for image detail and clarity. In a similar study, though not targeted toward patient audiences, Moin prompted two image generators (DALL-E and MIM) to create medical illustrations of four corneal transplant procedures; they found that images from all groups showed excellent detail and clarity.^21^ Additionally, Noel found DALL-E was able to create high quality, aesthetic images of the heart, brain, and skull.^4^ Unfortunately, image detail and clarity was the only criterion in which the image generators performed well; despite their visual appeal, images from all servers failed to produce medically accurate content. This has been shown in several other studies, who noted fictitious and/or grossly inaccurate anatomic structures portrayed in the AI-generated images (eg, duplicate appendages, foreign bodies, and pupils in improper locations).^4^^,^^21^^,^^27^

**Figure 8:**
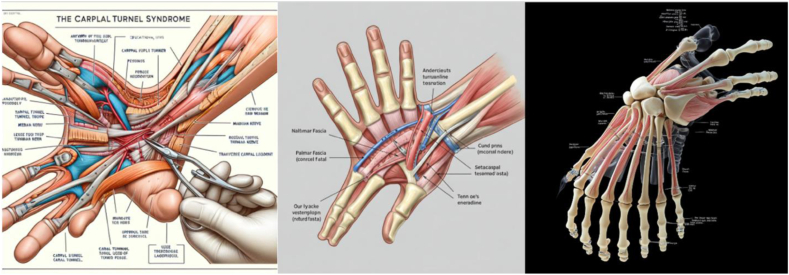
Example images containing significant fabrication. Left: DeepSeek image of CTS. Middle: Gemini image of Dupuytren contracture. Right: Midjourney image of thumb CMC arthritis.

Notably, 99.8% of images produced by the AI generators in our study included at least some fabricated anatomy. Although some images contained fictitious anatomy that did not detract from the overall image (eg, fabricated vessel, tendon, or small bony fragment), others had so many egregious structures that the image no longer resembled a human hand (Fig 8). The concept of fictitious structures parallels that of AI “hallucinations,” a well-documented limitation of AI models in which the server makes up information and references non-existent sources. For example, Tangrisivmol^28^ studied the use of Chat Generative Pre-trained Transformer (ChatGPT) in clinical medicine and found 40% of AI-generated discharge summaries contained hallucinations. Rawi^26^ also noted that ChatGPT commonly confabulated answers to frequently asked hand surgery questions and failed to provide citations for its clinical recommendations. Thus, the presence of illegitimate structures presents a major limitation to the use of AI in generating patient-facing resources.

Although we incorporated a large range of reputable AI image generators and achieved a large sample size, there are several limitations to our study. First, the queries were not iterative, ie, every search was generated in a new chat, and we did not provide the AI servers with our specific grading criteria. Although this was intended to allow each image generation to be unbiased by the previous ones, it in turn prevents the AI server from incorporating feedback to improve its outputs, as is seen deep learning processes.^29^ Second, individual images are not reproducible; each query resulted in a unique image that may greatly differ from the query before it. This could present challenges in standardization of images for mass-production of patient education materials. Finally, our images were evaluated by individuals with medical/surgical training; we did not account for patient interpretation or feedback on the efficacy of the images in portraying their surgical procedure. Future directions include providing more comprehensive inputs (references and objective criteria) to train AI models to create desired images and collecting patient feedback on such images.

In conclusion, although AI text-to-image generators have gained popularity in recent years, this study demonstrates significant limitations in the use of their images for patients undergoing surgery for common hand pathologies, such as CTS and TF. The AI generators were successful in producing images with excellent detail and clarity, but failed to depict accurate hand anatomy and appropriate labels. Moreover, greater than 99% of images contained at least some degree of fabricated anatomy, raising concern for their usability in patient settings.

Although AI offers many applications to hand surgery with the potential to improve the patient and provider experience, image generators are not currently able to create anatomically accurate materials for patient use. Further investigations should be performed on the ability to train AI models to generate images that incorporate accurate anatomy and appropriate labels prior to applying these images in patient-facing settings.

## Conflicts of Interest

Tamara D. Rozental, MD is a consultant for Stryker and Teladoc Health, Inc. There was no financial support or other benefits from commercial sources for the work reported in the manuscript, and the other authors have no relevant conflicts to disclose.
